# Multimodality MRI Findings in Patients with End-Stage Renal Disease

**DOI:** 10.1155/2015/697402

**Published:** 2015-05-04

**Authors:** Hui Juan Chen, Long Jiang Zhang, Guang Ming Lu

**Affiliations:** Department of Medical Imaging, Jinling Hospital, Medical School of Nanjing University, 305 Zhongshan East Road, Xuanwu District, Nanjing, Jiangsu 210002, China

## Abstract

Patients with end-stage renal disease (ESRD) suffer from a number of complex neurological complications including vascular damage and cognitive dysfunction. It is of great significance to detect the neurological complications and improve the prognosis of ESRD patients. Many new noninvasive MRI techniques have been steadily used for the diagnosis of occult central nervous system complications in ESRD patients. This gives an opportunity to understand the pathophysiological mechanisms of these neurological disorders. This paper is a review that presents the MRI findings of occult brain damage in ESRD patients, outlines the applications of advanced MRI techniques, and introduces a brief perspective in this study field.

## 1. Introduction

End-stage renal disease is now recognized to be a growing public health problem [1]. It has been estimated that 2-3% of the health care budget is spent on patients with end-stage renal disease, though these patients comprise only 0.1% of the general population [1]. The cost of care could be increased by cognitive impairment [2]. Cognitive impairment is commonly seen in individuals with chronic kidney disease (CKD), particularly among those treated with dialysis [3–6]. It is reported that the prevalence of cognitive impairment ranges from 16% to 38% in patients with ESRD [2]. The prevalence of moderate to severe cognitive impairment among hemodialysis or peritoneal dialysis patients was more than 2.5 times higher than that found in healthy controls [4]. Clinically, the patients with apparent neurological symptoms are likely to be paid more attention to, while those patients with cognitive deficit are often poorly recognized. This is not because these patients do not have cerebral abnormality but because cerebral abnormality is not detected in those patients.

Magnetic resonance imaging (MRI) is of great value for the diagnosis of apparent cerebrovascular damage. However, conventional MRI is limited in detecting neurological abnormality in ESRD patients with and without subtle vascular damage but with cognitive impairment. In the last decades, some advanced MRI techniques, such as diffusion weighted imaging (DWI), diffusion tensor imaging (DTI), and blood oxygen level dependent functional MRI (fMRI), have been widely used in the clinical and basic scientific studies. These advanced MRI techniques have been used to detect occult vascular damage, brain blood flow changes, brain atrophy, and brain functional connection in the resting state. This uncovers potential pathophysiological mechanisms of cognitive deficits in ESRD patients. This paper reviews the pathophysiological mechanisms of cognitive impairment, clinical presentations, and the potential applications of advanced MRI techniques in ESRD patients, especially focusing on patients without overt neurological complications. Last, a study perspective in this field is presented.

## 2. Pathophysiological Mechanisms of Cognitive Impairment

Though the cognitive decline is prevalent in patients with ESRD, its cause and pathophysiological mechanisms have not yet been clearly understood. More recently, researchers attributed the cognitive disorders and dementia to the disorders of the kidney-brain axis [3, 7, 8]. The brain and the kidneys share many common anatomic and vasoregulatory features and they are susceptible to vascular damage because of low vascular resistance system [9]. Three types of potential causes including traditional risk factors, nontraditional risk factors, and uremic toxins have been found to be associated with the vascular damage [3, 7]. In detail, traditional risk factors include ageing, hypertension, and diabetes mellitus. They mainly result in vascular injury. Nontraditional risk factors and uremic toxins are caused by the kidney damage. Nontraditional risk factors consist of chronic inflammation, oxidative stress, hypercoagulable state, and so forth. Uremic toxins include hyperhomocysteinemia, cystatin-C, and other factors caused by metabolic disorders. These latter two factors could lead to the vascular damage and endothelial dysfunction, even directly do damage to the neuron because of toxicity. Vascular damage and endothelial dysfunction finally result in cerebrovascular diseases such as stroke, white matter lesions, silent brain infarcts, and microbleeds. Dialysis may induce transient hypotension, arterial hypoxemia, and fluctuations in electrolytes and cerebral water content. These changes may lead to brain damage and finally result in ischaemic and haemorrhagic strokes [10]. In conclusion, the cognitive dysfunction in ESRD patients could be attributed to ESRD itself and treatment [11–13]. However, further research is needed to verify these hypotheses.

## 3. Clinical Presentations or Psychometric Assessment

In patients with ESRD, there is a wide range of diverse neurological complications such as white matter changes, cerebral atrophy, osmotic demyelination syndrome, dialysis encephalopathy, hypertensive encephalopathy, dementia, intracerebral hemorrhage, and opportunistic infections [12–14]. Patients with severe, acute complications may present typical symptoms such as abrupt headache, nausea, vomiting, consciousness alterations, myoclonus, tremor, and focal or generalized seizures. ESRD patients with chronic neurological complications often have cognitive function disturbance. Cognitive dysfunction includes reduced mental alertness, intellectual impairment, decreased attention and concentration, memory deficits, and diminished perceptual-motor coordination [15]. It is easily overlooked and ignored in its early stage. It is important to identify those patients with cognitive impairment to reduce the considerable morbidity associated with this condition and improve their life quality [16]. So far, a complete standardized psychometric test has not yet been established in patients with ESRD. Screening tests were proposed, that is, the mini-mental state exam (MMSE), Montreal Cognitive Assessment (MoCA), the Wechsler adult intelligence scale, the six-item screener, the clock drawing task, the mini-cog, trial making test part B, the digit span (D-span) psychometric test, and others [11, 15, 17, 18].

## 4. Magnetic Resonance Imaging

It has been reported that ESRD patients are inclined to develop neurological disorders such as white matter changes, cerebral atrophy, cerebral infarction, intracerebral hemorrhage, posterior reversible encephalopathy syndrome, osmotic demyelinization syndrome, cerebral infection, sinus vein thrombosis, and dialysis disequilibrium syndrome. Conventional MRI, which includes T_1_WI, T_2_WI, and fluid attenuated inversion recovery (FLAIR) images, is valuable for the diagnosis of these lesions [13, 14]. Detailed descriptions for MRI findings of these obvious neurological complications are beyond the scope of this review, and interested readers can read some excellent reviews elsewhere [13, 14].

Recently, some new MRI techniques have been widely used in the metabolic brain diseases. White matter microstructure changes, mild cerebral atrophy, cerebral microbleed, and brain function alterations can be detected with the advanced MRI techniques. These advanced MRI techniques are potential measures to investigate the occult neuropathological changes from brain structural and functional aspects in patients with ESRD. The following sections will introduce the applications of these advanced MR techniques used in the evaluation of brain damage in ESRD patients.

### 4.1. Brain Volume

Conventional MRI has high sensitivities for obvious cerebral atrophy, whereas it is limited in the diagnosis of early stage of cerebral atrophy. Voxel-based morphometry (VBM) is a whole-brain, unbiased technique for describing regional cerebral volume and tissue concentration differences in structural magnetic resonance images [19]. It can evaluate the microstructural changes of brain atrophy as well as its foci in early stage. In addition, the optimized VBM does better in resolving the problem of segmentation error than traditional VBM and produces more precise results by volume modulation [20]. Recently, VBM has become an effective tool to detect cortical atrophy and differences in slender white matter tracts [21].

**Figure 1:**
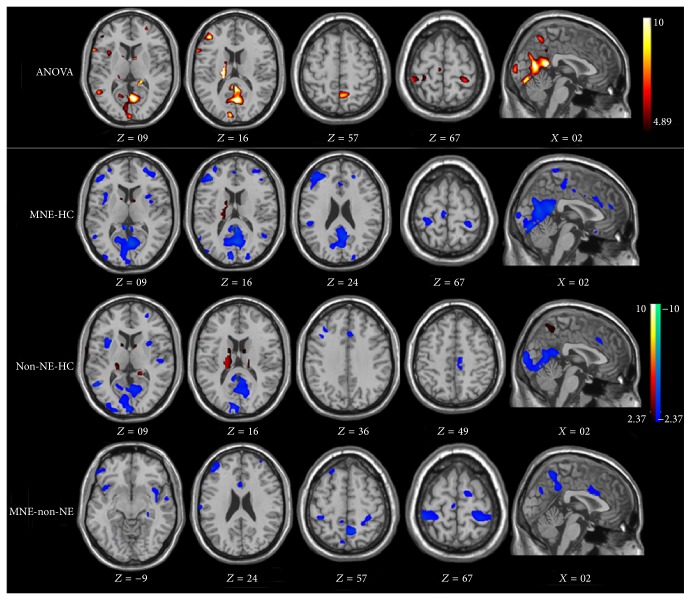
ANOVA test result and multiple comparisons of gray matter between groups. Compared with healthy controls, minimal nephroencephalopathy patients show decreased gray volume in the bilateral occipital lobes, precuneus/posterior cingulate cortex/cuneus, right frontal lobe, bilateral temporal pole, and left insula. Compared with healthy controls, nonnephroencephalopathy patients show decreased brain volume of bilateral occipital lobes, bilateral temporal poles, and posterior cingulate cortex/precuneus/cuneus and increased brain volume of right extra-nuclear, right caudate, and right thalamus. Compared with nonnephroencephalopathy patients, minimal nephroencephalopathy patients show decreased brain volume of right middle frontal gyrus, right postcentral gyrus, right occipital lobe, and left insula.

ESRD patients on hemodialysis show significant cerebral atrophy. This is correlated with longer hemodialysis duration and cognitive deficits. Prohovnik and his coworkers compared the global cerebral atrophy of ESRD patients on hemodialysis dialysis with healthy controls by VBM [11]. They found that patients with ESRD undergoing hemodialysis had significantly lower gray matter volume than healthy controls, particularly in the caudate nucleus. Moreover, these brain abnormalities are associated with longer hemodialysis duration and cognitive deficits. Zhang et al. [22] used VBM to investigate the pattern of brain volume changes in 57 patients with ESRD. It was found that, compared with healthy controls, ESRD patients showed diffusely decreased gray matter volume (Figure 1) and serum urea was negatively associated with changes, while dialysis duration was negatively associated with some white matter volume changes. This study suggested that uremic toxins might contribute to rapid brain atrophy, which might finally lead to cognitive decline. Qiu et al. [23] used VBM to characterize gray matter deficits in ESRD patients and found a significant decrease in gray matter volume in the bilateral medial orbitoprefrontal cortices, bilateral dorsal lateral prefrontal cortices, and the left middle temporal cortex in ESRD patients. Chai et al. [24] found that patients with haemodialysis showed decreased volume of bilateral putamen and left insular lobe compared with healthy controls. The decreased volume in the gray matter of left insular lobe positively correlated with MMSE scores suggesting that changes in the gray matter volume of the left insular lobe can account for the neurocognitive dysfunction in patients with haemodialysis. Additionally, dialysis duration was negatively associated with decreased volume of bilateral putamen which indicates that dialysis duration was also a risk factor for the brain atrophy [24]. In summary, VBM has been helpful in evaluating the cerebral atrophy and monitoring the progression of the disease.

### 4.2. Cerebral Microbleeds

Microbleeds may be a predictor of intracerebral hemorrhage, which is associated with cognitive dysfunction. It has been reported that the incidence of intracerebral hemorrhages in ESRD patients on hemodialysis has been approximately five to ten times higher than that in the general population [25]. Compared with nonhemodialysis patients, the mortality of hemodialysis patients with intracerebral hemorrhage is nearly twice as high [25]. Therefore, it has great implications for the detection of microbleeds in ESRD patients. However, small hemorrhage foci are often difficult to be detected on conventional MR images [26]. Compared with conventional T_2_WI sequences, T_2_∗WI is more sensitive in depicting small hemorrhage [20].

The incidence of microbleeds was found to be higher in the hemodialysis patients compared with the controls. In the study by Yokoyama et al. [25], eleven of the 57 hemodialysis patients had microbleeds on T_2_∗WI, while only two of the 53 control patients had microbleeds on T_2_∗WI. The total number of microbleeds in 11 hemodialysis patients was 44, whereas the control group had only two microbleeds. On T_2_- and T_1_WI only about 30% of microbleeds were shown as either hyperintensity or hypointensity, suggesting the higher sensitivity of T_2_∗WI. In another study by Watanabe [27], 80 patients who underwent maintenance hemodialysis were evaluated by MRI and 28 patients (35%, 28/80) had microbleeds in T_2_∗WI. This rate (35%) was higher than that of asymptomatic or healthy elderly individuals (about five to six percent). A correlation between microbleeds and intracerebral hemorrhage was found in this study. More microbleeds are associated with higher risk for intracerebral hemorrhage and cognition dysfunction. Therefore, T_2_∗WI should be used to detect microbleeds in ESRD patients.

**Figure 2:**
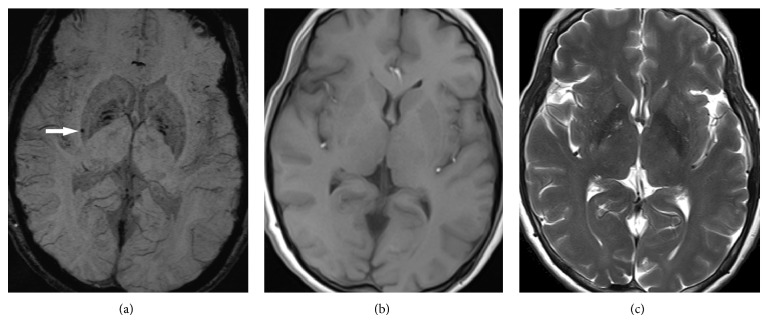
Magnetic resonance (MR) images of a 59-year-old man with end-stage renal disease. (a) SWI shows a hypointense lesion in the right putamen (arrow). (b) and (c) T1- and T2-weighted images do not show the corresponding lesion shown in (a).

Compared with T_2_∗WI, susceptibility-weighted imaging (SWI) can detect more microbleeds [28]. SWI is an MR technique that combines the use of both magnitude and phase images from a high-spatial resolution, three-dimensional GRE sequence [29]. One representative clinical case presented shows the advantages of SWI in the detection of microbleeds in ESRD patients with hemodialysis (Figure 2). However, further studies are needed to prove this hypothesis.

Another method, susceptibility mapping, which is sensitive to the subtle changes of iron content has been used in the studies of brain iron deposition in patients with haemodialysis. Susceptibility maps were reconstructed from original phase data. It can quantitatively calculate the susceptibility and the method has been shown to strongly correlate with iron concentration in the brain. With susceptibility mapping, Chai et al. found that the susceptibility of the bilateral caudate head, putamen, substantia nigra, red nucleus, and dentate nucleus in haemodialysis patients was higher than those in healthy controls, indicating that abnormal iron deposition did occur in patients with haemodialysis [30]. In a later study, Chai et al. found that increased brain iron deposition of bilateral putamen was a risk factor for the decreased gray matter volume of these two structures in patients with haemodialysis by using VBM and quantitative susceptibility mapping. However, these two studies are limited in small sample size and cross-sectional study. More studies with large cohort should be performed in the future [24].

### 4.3. Brain Edema and White Matter Damage

Diffusion weighted imaging (DWI) provides unique information about various pathological changes of the brain by depicting the microscopic movement of water molecules. The measurement of the water motion can be quantified absolutely by the introduction of the apparent diffusion coefficient (ADC) in DWI. The ADC mainly reflects the rate of diffusion in living systems. Diffusion tensor imaging (DTI) is an extension of DWI which could fully yield diffusion anisotropy effects, providing even more precise details on tissue microstructure [31]. With DTI, the assessment and visualization of large white matter fibers on a millimeter-level multidimensional scale become possible [32]. The fractional anisotropy (FA), relative anisotropy (RA), and the volume ratio indices are the most commonly used indices for the measurement of anisotropic diffusions by DTI. This combination of DTI and fiber tractography has provided an entirely new way of the evaluation of white matter connectivity in the human brain. These two techniques have greatly improved the understanding on neurologic and psychiatric disorders [33].

DWI may be an effective indicator to predict the prognosis in the ESRD patients. Kim et al. [34] reported a diabetic patient with uremia who was presented with acute bilateral basal ganglia lesions. These lesions appeared to have a high signal intensity on DWI and low diffusivity on the ADC map, especially in the globus pallidus. After a two-month treatment period, abnormal intensity in majority of the bilateral basal ganglia was normalized, except for the globus pallidus. This indicated that the ADC analysis might be helpful in estimating the prognosis of stroke in ESRD patients. However, more attention should be paid to the verdict as to the effectiveness of DWI regarding this application.

DWI is of great value to define the nature of edema, which is useful in explaining the pathogenesis of brain edema in ESRD patients. Kim et al. [34] reported that hyperintensities on DWI and decreased ADC values in the bilateral basal ganglia indicated cytotoxic edema during the acute phase. However, Lee et al. [35] as well as Yoon et al. [36] found that the increased ADC values were present in the basal ganglia of patients with diabetic uremia. These findings suggested that interstitial edema had occurred in these patients. Galons et al. [37] found that the brain edema induced by hemodialysis in uremic rats was due to interstitial edema rather than cytotoxic edema. Moreover, Chen et al. [38] found that brain ADC value in ESRD patients before and after hemodialysis increased significantly. These results suggested that edema was interstitial rather than cytotoxic in nature. Therefore, we could postulate that both edemas may coexist in uremic patients. Cytotoxic edema may be linked to the acute phase of nephroencephalopathy, while predominantly interstitial edema can occur in chronic phase of nephroencephalopathy.

**Figure 3:**
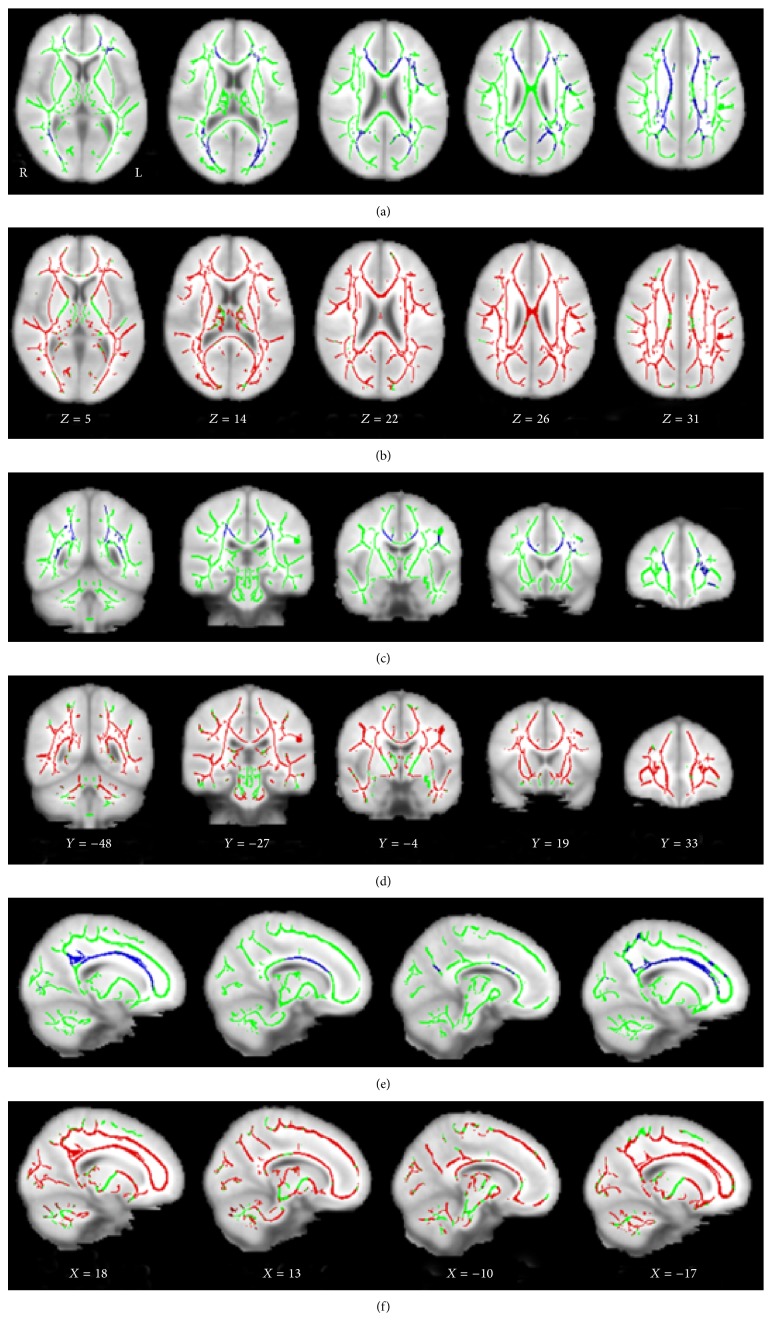
Significant altered brain regions with fractional anisotropy (FA) and mean diffusivity (MD) in hemodialysis ESRD patients compared with healthy controls. The background image is the standard MNI 152 brain template. Green voxels represent the white matter skeleton of FA. (a, c, and e) Blue voxels represent brain regions with reduced FA in hemodialysis ESRD patients (*P* < 0.05, FEW corrected). (a) Axial, (c) coronal, and (e) sagittal FA maps show the reduced FA regions are mainly located in the genu, splenium, and body of corpus callosum, bilateral anterior corona radiata, posterior corona radiata, superior corona radiata, and posterior thalamic radiation, left superior longitudinal fasciculus, and right cingulum. (b, d, and f) Red voxels represent brain regions with increased MD in hemodialysis ESRD patients (*P* < 0.05, FEW corrected). (b) Axial, (d) coronal, and (f) sagittal MD maps show the increased FA regions are located in almost all white matter regions and bilateral cerebellum, midbrain, and pons. FA: fractional anisotropy; MD: mean diffusivity; ESRD: end-stage renal disease; R: right; L: left.

DTI studies have demonstrated that ESRD patients have asymptomatic white matter damage. Hsieh et al. [39] found that ESRD patients had significantly lower FA values in all regions than was found in the control group by using manual region-of-interest analysis. FA values were generally lower in older patients and in those who had been undergoing dialysis for a longer duration. These might be correlated with axonal degeneration or demyelination of white matter. However, manual region-of-interest analysis lacks consistent standard for the size and position of the locus. The results are largely dependent on the locus and size of the region of interest drawn by the operators. In contrast, voxel-based analysis is an automatic method and it can provide more accurate results than manual region-of-interest analysis by the combination of normalizing whole-brain DTI to a standard coordinate system and utilizing diffeomorphic image registration. One voxel-wise DTI study [40] found that mean diffusivity (MD) and radial diffusivity (RD) representing the diffusivity in directions perpendicular to fiber orientations increased significantly in most brain regions in ESRD patients. Moreover, a positive correlation of MD and RD with the duration of hemodialysis suggests that hemodialysis may have gradually contributed to the demyelination of white matter tissue. This study also found that FA decreased significantly in patients with ESRD. The decreases in FA along with increases in MD were attributed to the reduced microstructural integrity with macroscopic tissue loss or interstitial edema. Long-term hemodialysis caused increased interstitial edema and gradually led to axonal demyelination in the pons. They believed that hemodialysis might have widespread effects on white matter in patients with ESRD. Recently, tract-based spatial statistics (TBSS) analyses, an unbiased whole-brain voxel-wise analysis of DTI data, was performed to investigate white matter alterations and their correlation with cognition function in ESRD patients undergoing hemodialysis [41, 42]. TBSS overcomes the voxel-based analysis disadvantages in smoothing and alignments. Consistent with previous studies, these studies found that ESRD patients had lower FA and higher MD values compared with healthy controls [41, 42] (Figure 3). They found that FA values showed correlations with neuropsychological tests, suggesting that cognitive impairment in ESRD patients may be associated with the microstructural abnormalities. Interstitial brain edema and white matter integrity disruption occurring in HD ESRD patients may account for the cognitive deficits in ESRD patients. However, the influence of peritoneal dialysis and renal transplantation on the white matter microstructures has not been reported yet.

DTI and fiber tractography have been used to investigate the pattern of brain white matter changes in patients with ESRD. Kim et al. [43] used diffusion tensor tractographies (DTTs) to investigate five neural tracts (corticospinal tract, fornix, superior longitudinal fasciculus, inferior longitudinal fasciculus, and inferior frontooccipital fasciculus) of four neurologically asymptomatic patients with ESRD. They discovered that all four patients had more than one lesion among the 10 DTTs, except for those of the corticospinal tract. This indicated that patients with ESRD had white matter fiber abnormalities on DTTs that were associated with cognition function. Further studies are needed for more and better clarification.

### 4.4. Brain Metabolism

Magnetic resonance spectroscopy (MRS) permits noninvasive evaluation of cerebral metabolites in vivo. It can provide specific information about the neurons, axons, the energy of cells, and the state of the membrane. Proton MRS (^1^H-MRS) has been widely used in the diagnosis of brain diseases as well as the understanding of neurological disorders. N-Acetylaspartate (NAA), choline-containing compounds (Cho), and myoinositol (mI), and so forth are closely related to the cerebral metabolic changes in ESRD patients.

**Figure 4:**
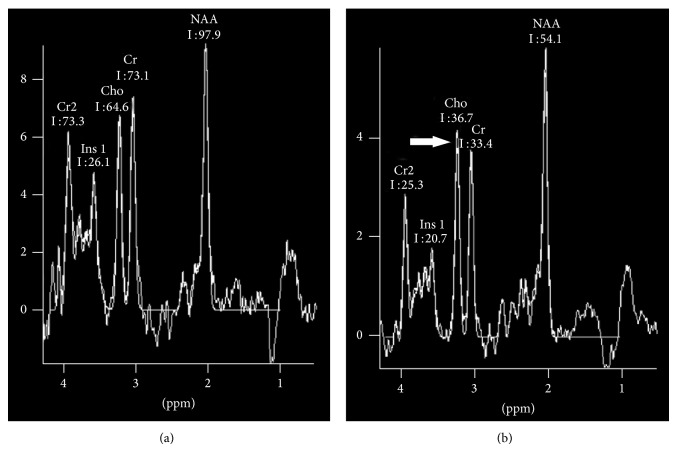
^1^H-MR spectroscopy findings in patient with end-stage renal disease and healthy subjects. (a) ^1^H-MR spectra in the left parietal white matter voxel in a 57-year-old healthy female. (b) ^1^H-MR spectra acquired in the right parietal white matter voxel in a 32-year-old healthy male with end-stage renal disease on peritoneal dialysis show elevated choline peak (arrow).

^1^H-MRS is useful in the detection of early changes in these metabolites in patients with ESRD. Most studies [44–48] reported an elevation of Cho/Cr (Figure 4), which is partly accompanied by the elevation of mI/Cr ratio in ESRD patients [45, 46]. As major osmolytes in the brain, the increase of Cho and mI could reflect the changes of osmotic pressure. Sasaki et al. [47] found that the increase of serum creatinine, blood urea nitrogen, and serum osmotic pressure usually led to the increase of cerebral Cho. Also, mI is considered as a glial marker. Chiu et al. [48] believed that the increased mI was related to either osmolytic changes associated with haemodialysis or gliosis. Therefore, the changes of Cho and mI may be used as biological markers in detecting early cerebral metabolic changes of ESRD patients. It remains contentious whether NAA is decreased in ESRD. Some studies [45, 49] found that NAA/Cr ratios were decreased in dialysed patients, while others [46–48] reported that the NAA/Cr ratios remained stable in the study population. The study populations, study designs, and the inherent shortcoming of the study method could account for these different results in these two studies. Until now, whether these metabolic disorders are associated with cognitive defects in ESRD patients remains unknown. Future studies should be performed.

^1^H-MRS is useful to monitor metabolic changes after treatment. Sasaki et al. reported that the Cho/Cr was reduced substantially after 18 months of hemodialysis [47]. This change suggested that hemodialysis could reverse the elevation of the cerebral Cho/Cr ratio. The elevation of the Cho/Cr ratio was an osmotic self-regulation of cerebral cellular membranes. However, the reduction of Cho/Cr was slow. No changes of Cho/Cr ratios were found after two weeks of initiating HD [47]. In summary, ^1^H-MRS could be used as a noninvasive imaging tool for monitoring the cerebral metabolic alterations in ESRD patients.

### 4.5. Cerebral Blood Flow

The cerebral blood flow (CBF) could be measured via dynamic susceptibility contrast and arterial spin-labeled perfusion (ASL) MRI. Dynamic contrast enhanced MR perfusion imaging uses gadolinium (Gd) as contrast agent which limits its use in ESRD patients. ASL technique utilizes arterial blood water as endogenous tracer by using radiofrequency irradiation. Without using contrast agents and radioactive tracer, ASL MRI measurements of cerebral blood flow have been validated against ^15^O-positron emission tomography (PET) in the brain [50]. Previous studies have found that ASL MRI was effective in assessing the cerebral blood flow in a variety of diseases such as acute ischemic stroke, brain tumors, and epilepsy [51]. With the above-mentioned advantage, ASL MR perfusion imaging has a potential to be used in patients with ESRD.

ASL MRI could be used as an effective means to investigate the cerebral blood flow changes in hemodialysis patients. One ASL MRI study by Prohovnik et al. [11] found that the cerebral blood flow of hemodialysis patients without anemia was reduced to 81% during the interdialytic cycle, and the blood flow of internal carotid artery was diminished and even more pronounced, about 60% of normal levels at the initiation of dialysis. However, the cerebral perfusion returned to normal levels during three to four hours of dialysis every two to three days after dialysis. Intriguingly, cognitive function is deteriorated during the interdialytic cycle and returns to an optimal level at around 24 hours after the haemodialysis session. Fluctuations of cognitive performance were found to be associated moderately with intradialytic hypotensive episodes [18]. Therefore, we speculated that certain association exists between cognition and the changes of cerebral blood flow. However, Regolisti et al. [52] found that the cerebral blood flow of patients with ESRD did not decrease during intermittent hemodialysis. Hence, further studies are needed to make it clear as to whether intermittent hemodialysis could decrease the cerebral blood flow in patients with ESRD and the underlying mechanism of this situation should be investigated.

### 4.6. Brain Function

Blood oxygen level-dependent (BOLD) signal has been accepted as the most widely used image contrast in functional MR imaging (fMRI) of the human brain [53] since its introduction in 1990. BOLD-fMRI is a well-established noninvasive technique for brain function study because it offers a great potential to uncover the neural mechanisms that underlie normal development, ageing, and neurological as well as psychiatric disorders [54]. BOLD-fMRI comprises resting-state and task-based fMRI. Task-based fMRI activation studies have several limitations such as long scan times, complicated study designs, and the great varieties of both subject and machine. In addition, it has difficulty in individual examination with cognitive impairment [55]. In contrast, resting-state fMRI is more feasible in patients without the necessity to complete the task. In resting-state fMRI, the participants are instructed to keep eyes closed and hold heads still and not to think of anything in particular during MR imaging scans lasting typically a few minutes. Resting-state fMRI not only enables the capability of better understanding on the pathophysiological process of the diseases but it also has the potential to discover the sensitive imaging biomarkers for early diagnosis of the neuropsychological diseases.

In the past years, much attention has been focused on detecting the brain activity in patients in the resting state and a few studies have been done on ESRD patients in a resting state using different analysis methods. Regional homogeneity (ReHo) is one of the algorithms to analyze the BOLD signal of the brain. It is proposed based on the hypothesis that the brain activity would more likely occur as clusters rather than as a single voxel; thus Kendall's coefficient of concordance (KCC) was used to evaluate the similarity between the time series of a given voxel and its nearest neighborhoods [56]. In one study by Liang et al. [57], ReHo was applied to investigate the pattern of spontaneous neural activity in 36 patients with ESRD. Diffused decreased ReHo values were found in both minimal nephroencephalopathy and nonnephroencephalopathy patients, which correlated well with neuropsychological impairments in these ESRD patients. Li et al. [58] found that ReHo values in neurologically asymptomatic patients with ESRD significantly increased in the bilateral superior temporal gyrus and left medial frontal gyrus but decreased in the right middle temporal gyrus. The results were a little different from the results by Liang et al., which might be related to the sample size. Despite this difference, the two studies suggested that changes of ReHo do take place in areas closely related to the memory and cognition, and the default mode network (DMN) might play an important role in the development of minimal nephroencephalopathy in ESRD patients.

**Figure 5:**
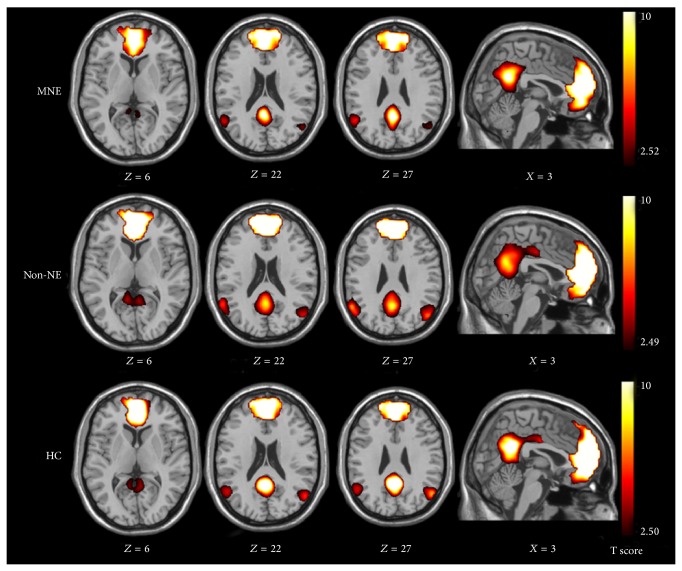
Spatial maps of default mode network (DMN) in minimal nephritic encephalopathy (MNE), nonnephritic encephalopathy (non-NE), and healthy control (HC) groups. DMN of the HCs consists of bilateral posterior cingulate cortex and adjacent precuneus, angular gyri, anterior cingulate cortex, medial frontal cortex, and superior temporal cortex (*P* < 0.05, false discovery rate corrected). A similar DMN but with some diffuse impairment of brain areas is shown on images of MNE and non-NE patients compared with those in HCs (*P* < 0.05, false discovery rate corrected).

**Figure 6:**
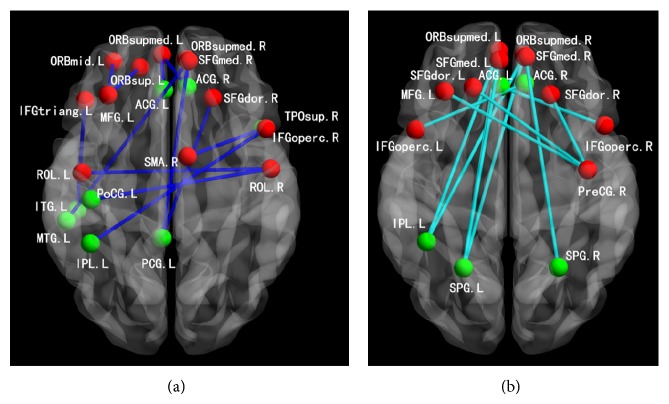
Decreased functional connectivity related with frontal lobes in ESRD patients. (a) Decreased positive connectivity in ESRD patients (blue edges). (b) Decreased negative connectivity in ESRD patients (light blue edges). Notes: red nodes: frontal lobe ROIs; green nodes: nonfrontal lobe ROIs; R: right; L: left.

Recently, with resting-state functional connectivity, several resting-state networks were revealed. The most studied network is the DMN. It typically comprises the posterior cingulate cortex (PCC), precuneus, rostral anterior cingulate, and medial prefrontal cortex (MPFC). An abnormal DMN has been found to be related to ESRD. A resting-state fMRI study by Ni et al. [59] demonstrated that the functional connectivity of DMN was impaired in patients with ESRD, especially in the PCC, precuneus, and MPFC regions. The results from the one-sample *t*-test showed a typical spatial pattern in the DMN for the healthy control subjects (HCs) in the resting state consisting of the bilateral PCC and adjacent precuneus, angular gyri, anterior cingulate cortex, MPFC, and superior temporal cortex (Figure 5) (*P* < 0.05, false discovery rate corrected). A similar DMN spatial pattern was observed in patients with ESRD (Figure 5). In patients with minimal nephroencephalopathy, the reduction of functional connectivity was even more pronounced in the MPFC, which might explain the reduced performance of these patients on neurocognitive tests. Therefore, it may be speculated that the DMN was impaired in this special population and the decline of cognition in these patients was well related to the changes of DMN. In another resting-state fMRI/VBM study by Qiu et al. [23] regions with gray matter volume reduction had significantly reduced resting-state functional connectivity with other brain regions. Recently, Zheng et al. [60] investigated the changes of full brain functional connectivity in ESRD patients with hemodialysis using resting-state fMRI and found widespread weakening of cortical and subcortical network connectivity in ESRD patients was more directly related to neuropsychological impairments and anemia rather than serum creatinine level, blood urea, and dialysis duration. In particular, impairments in the medial prefrontal lobe could play an important role in mediating psychological dysfunctions (Figure 6). In addition, Ma et al. [61] investigated the topological organization of functional brain networks in patients with ESRD by combining resting-state fMRI and graph-based approaches. This study demonstrated that patients exhibited significant disruption in parallel information processing over the whole networks. Moreover, the network abnormalities correlated with biochemical hemoglobin and serum calcium levels in the patients. These findings provide direct evidence for network disorganization in ESRD and would improve our understanding of ESRD from large-scale network perspective. In summary, these studies show a great potential of resting-state fMRI in uncovering the neuropathological mechanism of cognitive deficits in ESRD patients.

## 5. Perspectives

With the rapid development of MR imaging technique, a better understanding has been achieved concerning the pathophysiology of cognition impairment in patients with ESRD. However, there is still much to be done to improve on the diagnosis of minimal nephroencephalopathy (MMSE < 24) [50] with the advanced multimodality MRI techniques. Further demonstration of the exact neuropathological mechanism of cognitive dysfunction in ESRD patients needs to be studied. BOLD-fMRI should be used to further study and explain the neural mechanisms underlying the development of cognitive dysfunction. Most importantly, the effect of dialysis modality and renal transplantation on the brain function needs to be further clarified using multimodality structural and fMRI. Published fMRI studies in ESRD patients have been performed on a relatively small number of people; large-scale prospective studies need to be performed in the future. Additionally, multimodality MR imaging techniques, such as combination DTI and fMRI or MRI and PET (MRI/PET), should be investigated in the early detection of cognitive alterations in ESRD patients. Last but not least, more longitudinal studies should be performed to explore the patterns of brain structural and functional changes in these ESRD patients.

## 6. Conclusion

In conclusion, the advanced MRI is helpful in assessing the minimal neurological complications in patients with ESRD. With the application of multimodality MR techniques, further studies may uncover the neural mechanism underlying cognitive dysfunction in ESRD patients. Advanced MRI techniques are of great value in the early detection and diagnosis of minimal nephroencephalopathy (MMSE < 24). Thus, it would be helpful in evaluating the occult brain changes and improving both the morbidity and mortality of these ESRD patients.

## Abbreviations

ADC: Apparent diffusion coefficient
BOLD: Blood oxygen level dependent
DTI: Diffusion tensor imaging
DWI: Diffusion weighted imaging
ESRD: End-stage renal disease
fMRI: Functional magnetic resonance imaging
MRS: Magnetic resonance spectroscopy
VBM: Voxel-based morphometry.
